# Fasting blood glucose mediated the association between a body shape index and depression: a cross sectional study from NHANES 2017–2023

**DOI:** 10.3389/fnut.2025.1537644

**Published:** 2025-05-09

**Authors:** Yang-Tao Chen, Can-Jie Wei, Zhao-Chu Wang, Ya-Meng Xie, Xun Wang, Jing Wang

**Affiliations:** Department of Anorectal Surgery, The Affiliated People’s Hospital of Fujian University of Traditional Chinese Medicine, Fuzhou, China

**Keywords:** a body shape index, depression, fasting blood glucose, mediation effect, cross sectional study

## Abstract

**Objective:**

The objective was to evaluate ABSI’s association with depression and explore FBG as a possible mediating factor.

**Methods:**

Data from 8,748 NHANES participants (2017–2023) were analyzed. Logistic regression analyses assessed ABSI-depression associations, while mediation models tested FBG’s intermediary role. We conducted stratified analyses and interaction test to assess the impact of gender, age, race, PIR, education, alcohol use, current smoking status, BMI, hypertension history and hypercholesterolemia history on the study outcomes.

**Results:**

The fully adjusted logistic regression models demonstrated a significant positive association between ABSI and depression (OR = 1.20, 95%CI: 1.00, 1.44, *p* = 0.0497). Stratified analyses and interaction test showed that this association was significant only among participants with some college education or above (P for interaction < 0.05). No significant interactions were found across other subgroups. Mediation analyses revealed that FBG partially mediated the relationship between ABSI and depression (15.8%, *p* < 0.0001).

**Conclusion:**

ABSI was associated with depression, potentially mediated through FBG.

## Introduction

Depression, affecting over 280 million individuals globally, represents the foremost burden among mental health disorders and constitutes a critical public health challenge (1). Depression is a common mental health condition marked by enduring sadness, diminished interest or pleasure, and various cognitive and physical symptoms that can severely disrupt daily life (2, 3).

While researchers have extensively investigated conventional anthropometric measurements like Body Mass Index (BMI) and waist circumference in relation to depression (4–7) these metrics possess inherent limitations in capturing body composition complexity and its association with mental health outcomes. Specifically, BMI fails to differentiate between lean muscle mass and adipose tissue, offering limited insight into body fat distribution (8, 9). Recently, A Body Shape Index (ABSI) synthesizes non-linear correlations between height, weight, and waist parameters to predict mortality risk, as a morphological indicator (10). However, research examining ABSI’s association with mental health outcomes, particularly depression, remains limited. Understanding this relationship is vital, as body composition may influence mental health through various physiological mechanisms, including metabolic pathways (11–13).

Fasting blood glucose (FBG), a fundamental metabolic health marker, potentially mediates the association between body morphology and depressive symptomatology. Increasing evidence indicates that metabolic dysfunction, particularly insulin resistance (IR), plays a critical role in the pathophysiology and clinical features of depression, especially in the metabolic subtype of major depressive disorder (MDD). Individuals with treatment-resistant depression, especially those non-responsive to selective serotonin and norepinephrine reuptake inhibitors (SNRIs), tend to exhibit higher levels of IR and associated metabolic disturbances, including elevated insulin levels, body mass index, and fasting glucose. IR has been linked to core depressive symptoms such as, anhedonia, psychomotor changes, fatigue, and sleep disturbances, and it may contribute to poor antidepressant treatment response, particularly to SNRIs (14). Furthermore, depression itself may alter metabolic function through neuroendocrine and inflammatory signaling cascades, contributing to insulin resistance and hyperglycemia (15, 16), while depression itself may alter metabolic function such as insulin resistance (17, 18). Given the established correlation between fasting plasma glucose levels and anthropometric indices (19), the examination of FBG as an intermediary mechanism in the ABSI-depression pathway merits rigorous investigation.

To fill current research gaps, we evaluated whether FBG mediates ABSI’s impact on depression through population-based analysis. Specifically, we hypothesize that: (1) ABSI is positively associated with depressive symptoms; (2) FBG mediates this relationship, either partially or fully; and (3) these associations may vary across different demographic and health subgroups.

By employing mediation analysis techniques on the National Health and Nutrition Examination Survey (NHANES) cross-sectional data, our study seeks to elucidate the complex pathways linking ABSI to depression. Understanding these relationships could inform targeted interventions that consider both metabolic and psychological aspects of health, potentially leading to more effective strategies for depression prevention and treatment. Moreover, this research may provide new insights into the clinical utility of ABSI as a predictor of mental health outcomes and highlight the role of metabolic function in mental health status.

Our results may have important consequences for clinical practice and public health policy, potentially supporting the integration of body shape assessment and glucose monitoring in mental health screening and intervention programs. Additionally, this research may contribute to our understanding of the complex interplay between physical and mental health, suggesting new avenues for interdisciplinary approaches to health promotion and disease prevention.

## Materials, procedures, and analytical methods

### Research design and sample

NHANES is a widely recognized cross-sectional dataset that has been widely used in epidemiological research, conducted by the National Center for Health Statistics (NCHS) (20). It integrates surveys, physical assessments, and lab tests to evaluate the health and nutrition of both adults and children in the U.S. Since its inception in the early 1960s, NHANES has provided critical data on various health indicators, including chronic disease prevalence, risk factors, and lifestyle behaviors, enabling researchers and policymakers to track health trends, identify emerging health concerns, and guide public health interventions. The survey is designed to represent the non-institutionalized U.S. population and utilizes complex sampling techniques to ensure diversity across age, gender, race, and socioeconomic status, making its findings widely applicable to the general population.

**Figure 1:**
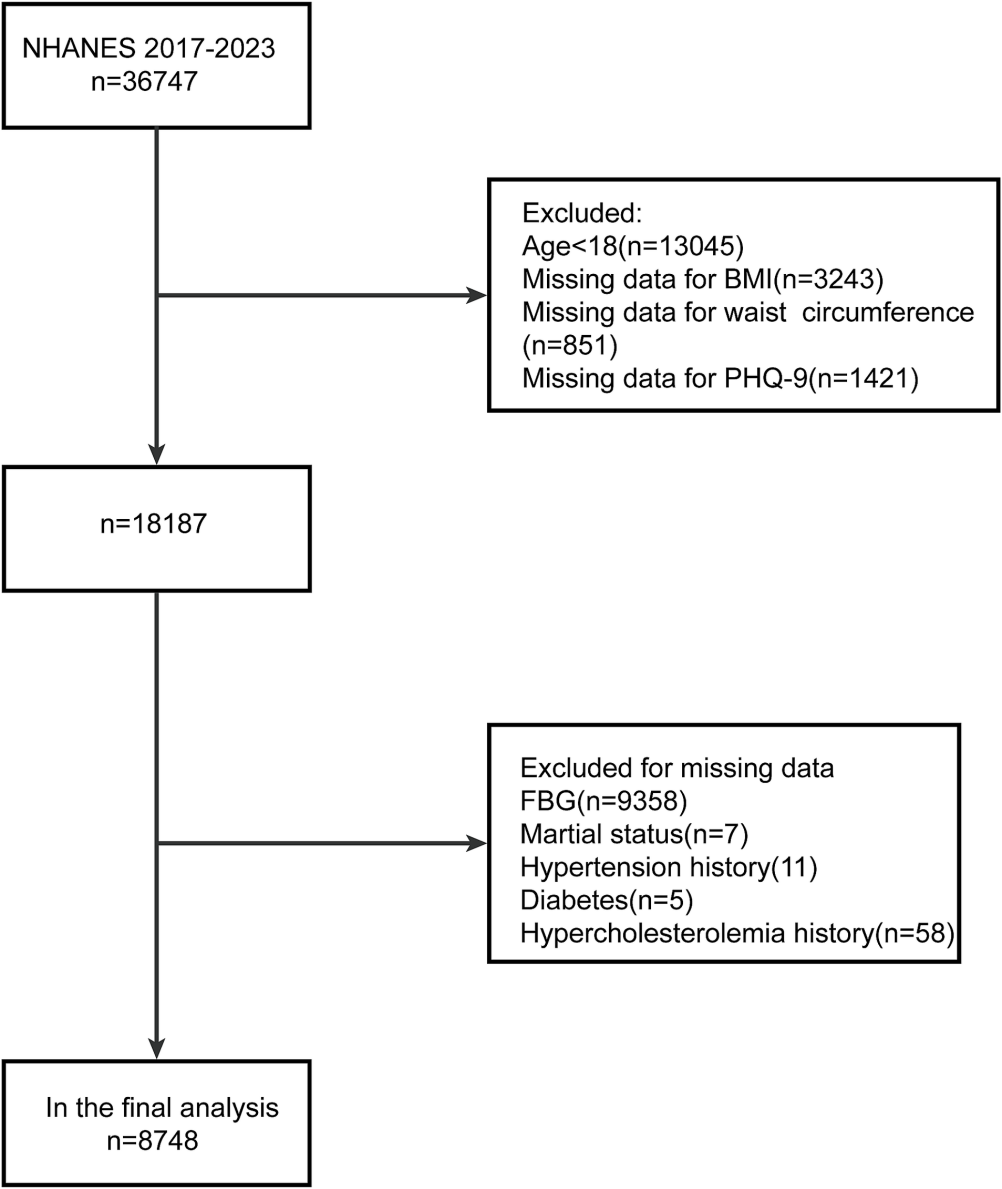
Participant selection flowchart from the 2017–2023 NHANES dataset. NHANES: National Health and Nutrition Examination Survey; BMI, Body Mass Index; PHQ-9, Patient Health Questionnaire-9; FBG, fasting blood glucose.

In this study, we utilized data from 2 NHANES cycles (2017–2020, 2021–2023), encompassing a total of 36,347 participants. After applying our inclusion and exclusion criteria, we retained a final analytic sample of 8,748 individuals, as illustrated in Figure 1.

### Data collection methods

Data collection was conducted by trained interviewers through household interviews and examinations at the mobile examination center (MEC). Standardized questionnaires administered during face-to-face interviews gathered demographic information (age, gender, race, marital status), behavioral factors (current smoking status, alcohol use), educational, poverty income ratio (PIR), medical history (hypertension, hypercholesterolemia, diabetes), and mental health condition as assessed by the Patient Health Questionnaire-9 (PHQ-9). Weight, height, and waist circumference were measured with calibrated digital scales and stadiometers to obtain anthropometric measurements. Blood samples collected at the MEC were analyzed for FBG at the central laboratory following standardized protocols (20).

### Index calculation

Body mass index (BMI) was calculated as body weight (kg) divided by height (m) squared. ABSI provides a better representation of abdominal fat distribution, which is more strongly linked to conditions like cardiovascular disease and type 2 diabetes (10). The formula for ABSI is:

$$\mathrm{ABSI}=\frac{WC}{BMI^{2/3}\times \mathrm{Heigh}t^{1/2}}$$

### Definitions

The PHQ-9 is a concise, self-administered questionnaire designed to assess the severity and presence of depressive symptoms (21). It is crucial to emphasize that PHQ-9 scores reflect depressive symptom burden rather than clinical diagnoses. The Patient Health Questionnaire-9 (PHQ-9) consists of nine items that assess symptoms related to major depressive disorder, including little interest, depressed mood, sleep issues, fatigue, changes in appetite, feelings of low self-worth, difficulty concentrating, changes in movement, and thoughts of suicide. Responses are rated on a scale from “not at all” to “nearly every day” over the preceding 2 weeks. Total scores range from 0 to 27, with scores ≥10 indicating clinically significant depressive symptoms; (22) therefore, in our study, participants were classified as “with depression” if their score was 10 or higher, and “without depression” if it was below 10.

BMI was used to assess obesity according to established categories: (1) normal weight: BMI between 18.5 and 24.9 kg/m^2^, (2) underweight: BMI of 18.4 kg/m^2^ or lower, (3) overweight: BMI between 25.0 and 29.9 kg/m^2^, and (4) obesity: BMI of 30.0 kg/m^2^ or higher (23). PIR was categorized as follows: low income (PIR ≤ 1.3) households (PIR ≤ 1.3), Low-to-middle-income households (>1.3, ≤3.5), and High-income households (PIR > 3.5). Alcohol use was categorized as frequent, occasional, rare, or seldom. Current smoking status include yes and not at all.

### Statistical analysis

Based on their PHQ-9 scores, participants were categorized into two groups. To address missing data in the NHANES dataset, we used a Random Forest (RF) algorithm for imputation. We employed either the Wilcoxon rank-sum test or the Kruskal-Wallis test to evaluate how continuous variables are distributed across various participant characteristics. For categorical variables, statistical significance was assessed using chi-square tests. Continuous variables are presented with either mean ± SD or median (IQR), and categorical variables are summarized by frequencies. Multiple logistic regression models were employed to analyze the relationship between ABSI and depressive symptom severity, as well as between FBG and depressive symptom severity, with results presented using odds ratios (OR) and corresponding confidence intervals. Multiple linear regression analysis was used to examine the association between ABSI and FBG. Three models were evaluated: (1) Model I: no adjustments; (2) Model II: adjusted for age, gender, and race; and (3) Model III: adjusted for age, gender, race, education, PIR, marital status, alcohol use, smoking status, hypertension history, and hypercholesterolemia history. Based on the fully adjusted Model III, we further explored potential effect modifications by conducting stratified and interaction analyses across subgroups defined by gender, age, race, PIR, education, alcohol use, current smoking status, BMI, hypertension history and hypercholesterolemia history. The findings were illustrated using forest plots for clarity and comparison. Due to the small range of the original variable ABSI (values typically below 1), the variable was rescaled by multiplying by 100 to enhance the interpretability of the regression coefficients. The new scale represents changes per unit increase, equivalent to 0.01 increments in the original variable (ABSI).

To assess the extent of mediation by FBG in the relationship between ABSI and depression causal mediation analyses were conducted. The “mediator” R package was used to estimate the direct, indirect, and total effects (24). Mediation analysis seeks to decompose the overall effect of an exposure into both direct and indirect effects (25). In this study, our goal is to decompose the total effect of depression into two components: “one mediated by the ABSI-related risk of elevated FBG (indirect effect) and the other directly attributed to ABSI (i.e., not mediated through fasting blood glucose). Mediation analyses were conducted using the Mediation package alongside the PROCESS macro, with bootstrap estimates of confidence intervals for the mediating effect and adjustments based on Model III to evaluate the proportion of the effect mediated by fasting blood glucose. A *p*-value of less than 0.05 was considered statistically significant, with values below this cut-off indicating significant results.

All statistical analyses were performed using R software (version 4.4.2) and EmpowerRCH (version 4.2), with figures created in Adobe Illustrator 2024.

## Results

### Characteristics of the participants

A large-scale investigation encompassing 8,748 subjects utilized PHQ-9 scoring to categorize participants into two cohorts: 8,018 individuals without depressive symptoms and 730 with depression. Analysis revealed that while ABSI measurements remained comparable between groups, BMI-defined obesity was more prevalent among depressed individuals (BMI ≥ 30 kg/m^2^). The study identified a gender disparity, with women showing higher depression rates than men. Additionally, the findings suggested that depression was associated with increased fasting blood glucose levels in this study population. Table 1 presents the baseline characteristics.

**Table 1:** Characteristics of the study population.

	Without depression (*n* = 8,018)	With depression (*n* = 730)	*p* value
Age	50.73 ± 18.10	48.18 ± 17.51	<0.001
ABSI	0.08 ± 0.01	0.08 ± 0.01	0.358
FBG (mmol/L)	6.16 ± 1.89	6.55 ± 2.65	<0.001
BMI (kg/m^2^)			<0.001
< 25	2,249 (28.05%)	153 (20.96%)	
≥ 25,<30	2,579 (32.17%)	203 (27.81%)	
≥ 30	3,190 (39.79%)	374 (51.23%)	
Waist circumference (cm)	99.93 ± 16.90	104.35 ± 19.17	<0.001
Height (cm)	167.32 ± 9.89	166.13 ± 10.08	0.002
Gender			<0.001
Male	3,932 (49.04%)	266 (36.44%)	
Female	4,086 (50.96%)	464 (63.56%)	
Race			0.029
Mexican American	935 (11.66%)	83 (11.37%)	
Other Hispanic	759 (9.47%)	91 (12.47%)	
Non-Hispanic White person	3,491 (43.54%)	307 (42.05%)	
Non-Hispanic Black person	1,576 (19.66%)	155 (21.23%)	
Other Race	1,257 (15.68%)	94 (12.88%)	
PIR			<0.001
≤ 1.30	1745 (21.76%)	269 (36.85%)	
> 1.3, ≤3.5	3,424 (42.70%)	313 (42.88%)	
> 3.5	2,849 (35.53%)	148 (20.27%)	
Education			<0.001
Less than 9th grade	450 (5.61%)	66 (9.04%)	
9-11th grade	742 (9.25%)	101 (13.84%)	
High school graduate	1899 (23.68%)	183 (25.07%)	
Some college	2,566 (32.00%)	266 (36.44%)	
College graduate or above	2,361 (29.45%)	114 (15.62%)	
Marital status			<0.001
Married/Living with partner	4,449 (55.49%)	265 (36.30%)	
Widowed/Divorced/Separated	1,385 (17.27%)	182 (24.93%)	
Never married	1,406 (17.54%)	198 (27.12%)	
Alcohol use			0.003
Frequent	2,596 (32.38%)	285 (39.04%)	
Occasional	1854 (23.12%)	157 (21.51%)	
Rare	1722 (21.48%)	133 (18.22%)	
Seldom	1846 (23.02%)	155 (21.23%)	
Current smoking status			<0.001
Yes	2,969 (37.03%)	357 (48.90%)	
Not at all	5,049 (62.97%)	373 (51.10%)	
Hypertension history			<0.001
Yes	2,856 (35.62%)	346 (47.40%)	
No	5,162 (64.38%)	384 (52.60%)	
Hypercholesterolemia history			0.107
Yes	2,987 (37.25%)	294 (40.27%)	
No	5,031 (62.75%)	436 (59.73%)	
Diabetes history			
Yes	1,105 (13.78%)	168 (23.01%)	
No	6,665 (83.13%)	538 (73.70%)	
Borderline	248 (3.09%)	24 (3.29%)	

Without depression, PHQ-9 score<10; With depression, PHQ-9 score≥10; ABSI, A body shape index; PIR, poverty income ratio.

### Relationship between ABSI, fasting blood glucose and depression

Table 2 presents the findings from multiple logistic regression analyses examining the relationship between ABSI and depression. Our analysis revealed an interesting pattern regarding the relationship between ABSI and depression across different models. While Model I showed no meaningful relationship between ABSI and depression (OR = 1.07, 95% CI: 0.92, 1.25, *p* = 0.3575), after adjusting for potential confounders in Model III, we observed a significant relationship. Specifically, each 0.01-unit increment in ABSI was associated with a 20% higher risk of depression (OR = 1.220, 95%CI: 1.23, 1.75, *p* = 0.0497).

**Table 2:** Multiple logistic regression result relationship between ABSI and depression.

	Model I OR (95%CI) *p* value	Model II OR (95%CI) *p* value	Model III OR (95%CI) *p* value
ABSI	1.07 (0.92, 1.25) 0.3575	1.47 (1.23, 1.75) < 0.0001	1.20 (1.00, 1.44) 0.0497

Model I: no adjustments; Model II: adjusted for age, gender, and race; Model III: adjusted for age, gender, race, education, poverty-to-income ratio (PIR), marital status, alcohol use, smoking status, and hypercholesterolemia history.

**Figure 2:**
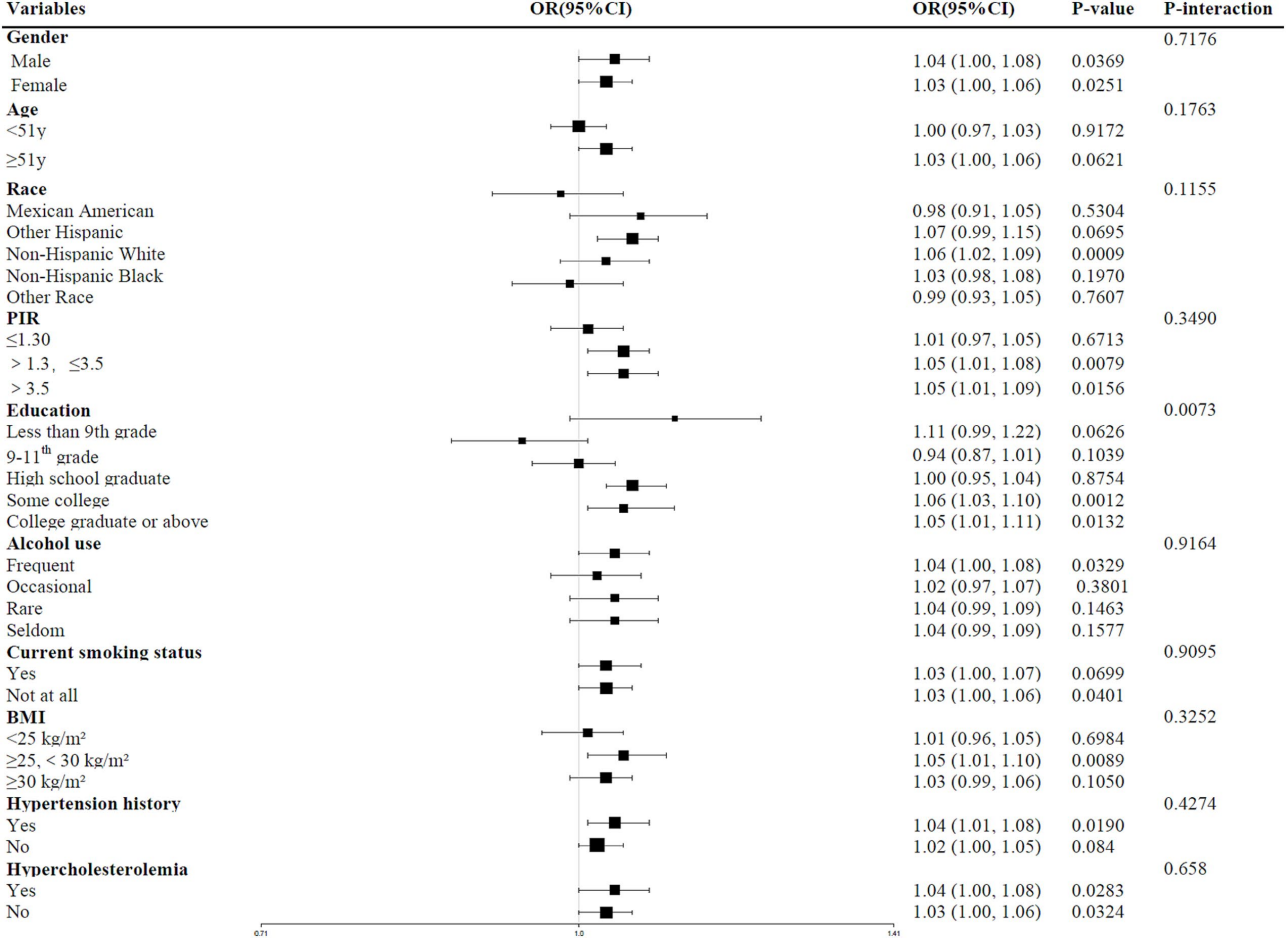
Stratified analysis and interaction effects of ABSI on depression across subgroups.

In the stratified analyses, a statistically significant interaction was observed between ABSI and education level in relation to depression (P for interaction < 0.05). Specifically, the association between higher ABSI and increased odds of depression was significant only among participants with some college education or above (*p* < 0.05). No significant interactions were found across other subgroups, including gender, age, race, PIR, alcohol use, current smoking status, body mass index (BMI), history of hypertension, or history of hypercholesterolemia (all P for interaction > 0.05), suggesting that the modifying effect of ABSI on depression risk was consistent across these groups. More details in Figure 2.

Additionally, we investigated the associations between ABSI and FBG, as well as the relationship between FBG and depression. Under Model III adjustments, a significant positive correlation emerged between ABSI and FBG (*β* = 1.08, 95%CI: 1.05, 1.12, *p* < 0.0001). Similarly, elevated FBG levels were positively associated with depression (β = 0.02, 95%CI: 0.01, 0.02, *p* < 0.0001) (Table 3).

**Table 3:** Relationships Between FBG and Depression, and Between ABSI and FBG.

	Model I *β* (95%CI) *p* value	Model II β (95%CI) *p* value	Model III β (95%CI) *p* value
FBG and depression			
Without depression	Ref	Ref	Ref
With depression	1.08 (1.05, 1.11) < 0.0001	1.11 (1.08, 1.14) < 0.0001	1.08 (1.05, 1.12) < 0.0001
ABSI and FBG	0.05 (0.05, 0.06) < 0.0001	0.02 (0.02, 0.03) < 0.0001	0.02 (0.01, 0.02) < 0.0001

Model I: no adjustments; Model II: adjusted for age, gender, and race; Model III: adjusted for age, gender, race, education, poverty-to-income ratio (PIR), marital status, alcohol use, smoking status, and hypercholesterolemia history.

### Mediation analysis

Mediation analyses were conducted to evaluate the extent to which FBG mediates the relationship between ABSI and depression. The results indicated a statistically significant indirect effect of ABSI on depression via FBG (Effect = 0.002, *p* < 0.001). In contrast, the direct effect of ABSI on depression was not significant (Effect = 0.009, *p* = 0.06). The total effect was significant (Effect = 0.01, *p* = 0.03), with the indirect effect contributing 15.8% of the overall association (Figure 2).

## Discussion

**Figure 3:**
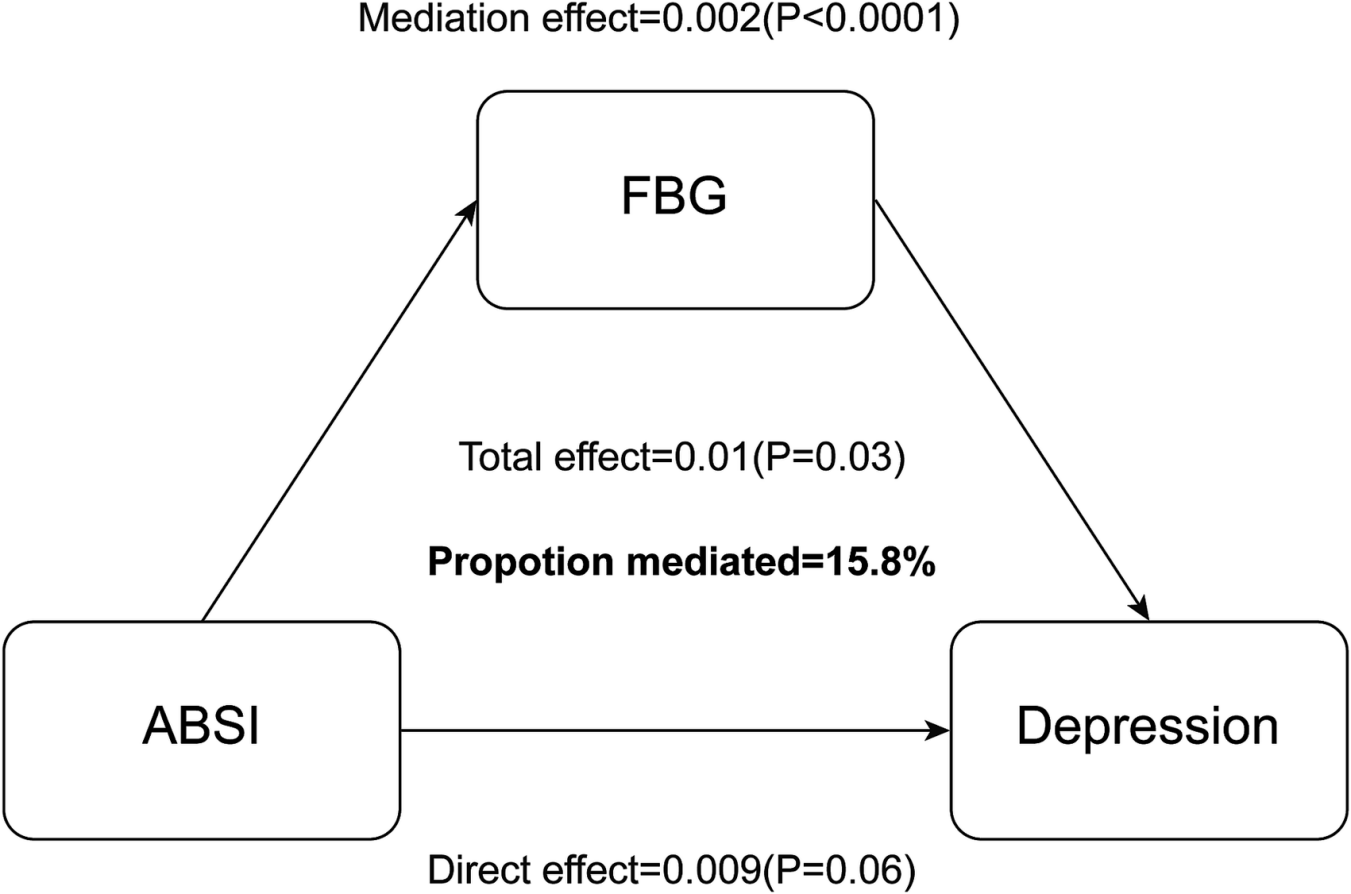
FBG serves as a mediator in the relationship between ABSI and depression, influencing the effect between these two variables.

This cross-sectional investigation analyzed data from the NHANES database (2017–2023), encompassing 8,748 participants. The research examined the relationship between ABSI and depression, while evaluating the mediating role of FBG. After implementing comprehensive covariate adjustments, analysis revealed that each 0.01-unit increment in ABSI was associated with a 20% increased prevalence of depression. Significant positive associations emerged among all variables: between ABSI and depression, between FBG and depression, and between ABSI and FBG. Notably, FBG showed a significant mediating role in the connection between ABSI and depression (see Figure 3).

However, in the relationship between ABSI and depression, the discrepancy in statistical significance between the unadjusted model (Model I) and the adjusted models (Models II and III) suggests the presence of confounding variables. In the unadjusted model, the effect of ABSI on depression may have been obscured by demographic and health-related factors. After adjusting for age, gender, and race in Model II, and further controlling for socioeconomic status, BMI, and clinical factors in Model III, a significant association emerged. This indicates that ABSI is independently associated with depression when potential confounders are taken into account. Notably, factors such as BMI, PIR, and comorbidities like hypertension and hypercholesterolemia may play a role in masking or modifying the true relationship between ABSI and depressive symptoms in the crude model.

The observed interaction between ABSI and education level suggests that educational attainment may modify the relationship between body shape and depression. One possible explanation is that individuals with higher education may be more aware of body image and health-related risks associated with central adiposity, potentially increasing psychological vulnerability. Alternatively, greater health literacy among the more educated may lead to increased detection or reporting of depressive symptoms. The absence of significant interactions in other subgroups indicates that the association between ABSI and depression is generally stable across demographic and clinical characteristics.

The significant association between ABSI and depression observed in our study can be explained through several potential mechanisms: From a physiological standpoint, central adiposity, as indicated by elevated ABSI values, triggers a state of chronic inflammation that promotes the secretion of pro-inflammatory cytokines, including interleukin-6 (IL-6), C-reactive protein (CRP), (26) and tumor necrosis factor-alpha (TNF-*α*). These inflammatory mediators can penetrate the blood–brain barrier, disrupting neurotransmitter metabolism, particularly serotonergic system function, which consequently elevates the risk of depression (27). Additionally, excessive adiposity can lead to decreased glutamine levels, potentially contributing to depressive symptoms (28). Furthermore, elevated ABSI is associated with visceral fat accumulation, which can disrupt hypothalamic–pituitary–adrenal (HPA) axis function (29). This disruption alters cortisol secretion patterns, (30) directly impacting mood regulation and potentially exacerbating cognitive decline, thereby creating a self-perpetuating cycle (31). The extensive neural connections between the hypothalamus and key brain regions, including the hippocampus, amygdala, and cortical areas, underscore its crucial role in cognitive function and emotional regulation (32). Conversely, dysregulation of HPA axis stress responsivity may contribute to the development of obesity (29). From a psychosocial perspective, elevated ABSI values often correlate with negative body image perceptions, which can diminish self-esteem and promote social isolation. Individuals with higher body weight frequently face societal stigmatization, being unfairly stereotyped as lacking willpower, self-discipline, and motivation (33). This internalized stigma and subsequent alterations in social interaction patterns may exacerbate depressive symptomatology. These negative emotional states can trigger maladaptive eating behaviors, such as binge eating, further contributing to weight gain (33) and establishing a cyclical pattern of psychological distress, obesity, stigmatization, and increased stress (34). The connection between ABSI and depression seems to be reciprocal: depressive episodes frequently precipitate modifications in dietary patterns and lifestyle behaviors, including hyperphagia and physical inactivity, which can subsequently alter body composition and elevate ABSI values (35, 36).

Our study demonstrated a positive correlation between ABSI and FBG, consistent with well-established mechanisms linking obesity and glucose regulation. ABSI serves as an indicator of central adiposity, characterized by excessive visceral fat accumulation. This metabolically active visceral tissue secretes free fatty acids (FFAs) and their metabolites, including acyl-coenzyme A, ceramides, and diacylglycerol, which impair cellular insulin responsiveness, leading to insulin resistance and elevated FBG levels (37). Central adiposity also triggers chronic low-grade inflammation, where enlarged, dysfunctional adipocytes secrete pro-inflammatory cytokines, particularly tumor necrosis factor-*α* (TNF-α). These inflammatory mediators disrupt insulin signaling pathways and compromise pancreatic *β*-cell function, exacerbating hyperglycemia (38). Furthermore, central adiposity disrupts hormonal homeostasis by altering leptin and adiponectin secretion patterns. The resultant leptin resistance and reduced adiponectin levels significantly impair glucose metabolism (39, 40). Additionally, central obesity-associated elevation in cortisol levels enhances hepatic glucose production, further contributing to increased FBG levels (30).

We confirmed the association between FBG and depression, warranting further investigation into the underlying mechanisms. Elevated FBG levels are characterized by chronic low-grade inflammation, where pro-inflammatory cytokines, particularly TNF-*α*, penetrate the blood–brain barrier and activate microglial cells within the central nervous system (41). This neuroinflammatory cascade disrupts neurotransmitter homeostasis, specifically affecting serotonin and dopamine synthesis and metabolism, which are essential for mood regulation. Notably, inflammatory processes shift tryptophan metabolism toward the kynurenine pathway, diminishing serotonin availability and consequently increasing depression susceptibility (42). Persistent hyperglycemia generates oxidative stress and promotes advanced glycation end products (AGEs) accumulation, resulting in neurotoxicity. These pathophysiological changes induce neuronal damage and synaptic dysfunction, particularly affecting the prefrontal cortex and amygdala (43–45)—regions crucial for emotional processing. The resultant structural and functional alterations in these brain regions manifest as core depressive symptoms, including emotional dysregulation and cognitive impairment (46, 47).

Our mediation analysis revealed that FBG partially mediates the relationship between ABSI and depression, accounting for 15.8% of the total effect. This finding highlights the complex interplay between body composition, glucose metabolism, and mental health, which suggests a parallel physiological route through metabolic dysfunction.

The identification of FBG as a partial mediator has important clinical implications. It suggests that interventions targeting glucose metabolism might have beneficial effects on mental health in individuals with elevated ABSI. Conversely, depression management in individuals with central obesity should include careful monitoring of glucose metabolism. This integrated approach could potentially break the cycle of metabolic dysfunction and psychological distress. However, certain limitations should be acknowledged. The cross-sectional nature of our study prevents us from establishing temporal relationships between variables. While our findings support FBG as a mediator, longitudinal studies are needed to confirm the causal pathways. Additionally, the 15.8% mediation effect suggests that other mediators likely exist, warranting investigation into additional biological and psychological pathways.

## Conclusion

This study demonstrates that FBG partially mediates the relationship between ABSI and depression, accounting for 15.8% of the total effect. These findings suggest the importance of monitoring glucose metabolism in individuals with elevated ABSI for comprehensive depression management.

## Data Availability

The original contributions presented in the study are included in the article/Supplementary material, further inquiries can be directed to the corresponding author.
